# An Isothermal Molecular Point of Care Testing for African Swine Fever Virus Using Recombinase-Aided Amplification and Lateral Flow Assay Without the Need to Extract Nucleic Acids in Blood

**DOI:** 10.3389/fcimb.2021.633763

**Published:** 2021-03-17

**Authors:** Yuhang Zhang, Qingmei Li, Junqing Guo, Dongliang Li, Li Wang, Xun Wang, Guangxu Xing, Ruiguang Deng, Gaiping Zhang

**Affiliations:** ^1^ College of Veterinary Medicine, Henan Agricultural University, Zhengzhou, China; ^2^ Key Laboratory of Animal Immunology, Henan Academy of Agricultural Sciences, Zhengzhou, China; ^3^ Jiangsu Co-Innovation Center for Prevention and Control of Important Animal Infectious Disease and Zoonose, Yangzhou University, Yangzhou, China

**Keywords:** point of care testing, African swine fever, isothermal molecular diagnosis, recombinase-aided amplification, lateral flow assay, blood

## Abstract

African swine fever (ASF) is a highly contagious and usually deadly porcine infectious disease listed as a notifiable disease by the World Organization for Animal Health (OIE). It has brought huge economic losses worldwide, especially since 2018, the first outbreak in China. As there are still no effective vaccines available to date, diagnosis of ASF is essential for its surveillance and control, especially in areas far from city with limited resources and poor settings. In this study, a sensitive, specific, rapid, and simple molecular point of care testing for African swine fever virus (ASFV) *B646L* gene in blood samples was established, including treatment of blood samples with simple dilution and boiling for 5 min, isothermal amplification with recombinase-aided amplification (RAA) at 37°C in a water bath for 10 min, and visual readout with lateral flow assay (LFA) at room temperature for 10–15 min. Without the need to extract viral DNA in blood samples, the intact workflow from sampling to final diagnostic decision can be completed with minimal equipment requirement in 30 min. The detection limit of RAA-LFA for synthesized *B646L* gene-containing plasmid was 10 copies/μl, which was 10-fold more sensitive than OIE-recommended PCR and quantitative PCR. In addition, no positive readout of RAA-LFA was observed in testing classical swine fever virus, porcine reproductive and respiratory syndrome virus, porcine epidemic diarrhea virus, pseudorabies virus and porcine circovirus 2, exhibiting good specificity. Evaluation of clinical blood samples of RAA-LFA showed 100% coincident rate with OIE-recommended PCR, in testing both extracted DNAs and treated bloods. We also found that some components in blood samples greatly inhibited PCR performance, but had little effect on RAA. Inhibitory effect can be eliminated when blood was diluted at least 32–64-fold for direct PCR, while only a 2–4 fold dilution of blood was suitable for direct RAA, indicating RAA is a better choice than PCR when blood is used as detecting sample. Taken together, we established an sensitive, specific, rapid, and simple RAA-LFA for ASFV molecular detection without the need to extract viral DNA, providing a good choice for point of care testing of ASF diagnosis in the future.

## Introduction

African swine fever (ASF) is an infectious disease of domestic pigs and wild boars of all breeds and ages, usually showing symptoms like high fever and hemorrhages with a high mortality rate. It endangers swine industries and brings huge economic losses each year, and thus is listed as a reportable disease by the World Organization for Animal Health (OIE) (Penrith and Vosloo, 2009). African swine fever virus (ASFV), the causative agent of ASF, is a large enveloped double-stranded DNA virus. It is the only member of the *Asfarviridae* family, *Asfivirus* genus, with a large viral genome around 170–194 kb, encoding at least 125 viral proteins (Dixon et al., 2013). Based on sequencing of the 3’ terminal end of the *B646L* that encodes the p72 protein major capsid protein, ASFV can be divided up to 24 distinct genotypes (I-XXIV), of which only genotypes I and II have been found outside of African continent (Bastos et al., 2003; Quembo et al., 2018). ASFV is extremely resistant to heat, desiccation, putrefaction, and varified pH conditions, especially in environments with proteins and low temperature, making ASF a highly contagious disease that can be transmitted not only by infected pigs or tampans, but also by contaminated materials such as meat, blood, feces, urine, or saliva from infected pigs (Brown and Bevins, 2018). ASFV can even remain infectivity over 75 weeks at 4°C in blood (Plowright and Parker, 1967). High stability of ASFV makes eradication and prevention of ASF a challenging work during live pig production.

ASF was first reported in Kenya in 1921 and circulated only in Sub-Saharan Africa for decades. In 1957, an outbreak of ASF from African continent invaded Portugal and Spain, resulting in an ASF epidemic within European countries until its eradication in 1990s. In 2007, another outbreak of ASF started from Georgia and rapidly spread across Eastern Europe (Costard et al., 2013). The first case of ASF in China was reported on August 3, 2018 (Zhou et al., 2018). Since then, ASF cases were continuously reported from all provinces of China and neighboring Asian countries, including Mongolia (January 2019), Vietnam (February 2019), Cambodia (March 2019), Korea (May 2019), Laos (June 2019), Myanmar (August 2019), Philippines (July 2019), Timor-Leste (September 2019), Indonesia (November 2019), Papua New Guinea (March 2020), and India (May 2020). To date, ASF is mainly distributed in Africa, Europe, and Asia, revealing a serious deterioration. ASF has led to a total of 8,202,702 pigs lost since 2018, of which Asia accounts for 82% (OIE, 2020).

Though efforts have been put in developing ASF vaccines with varying levels of success, there is still no vaccine available to date (Bosch-Camos et al., 2020). Control and prevention of ASF are mainly based on animal slaughter and strict sanitation strategies, in the aid of early detection and surveillance of the disease. However, sensitive, specific clinical diagnosis of ASF is hard to make, due to its non-specific symptoms which are similar to some common porcine diseases such as classical swine fever. Traditional OIE recommended diagnostic methods for ASF include virus isolation, antigen identification (hemadsorption test and fluorescent antibody test) and serological tests (enzyme-linked immunosorbent assay, indirect fluorescent antibody test, indirect immunoperoxidase test and immunoblotting test) (OIE, 2019). All these methods need to be performed by skilled technicians with expensive equipments under laboratory condition, often leading to diagnosis delay and virus transmission, especially in areas far from city with limited resources and poor settings.

With a short incubation time from 4 to 19 days, some ASF cases even show no clinical signs and antibody responses before pig death and virus spread. In addition, ASFV genomic DNA can be detected in blood as early as 56 h post-infection (Wang X. et al., 2020) and stably exist up to 78 days (Gallardo et al., 2019), making it an ideal choice for ASFV early and bioptic molecular detection. Current ASFV molecular detection methods used in laboratory mainly rely on OIE recommended PCR and real-time PCR. However, it is well known that some components in blood have the inhibitory effects on PCR, which limits its performance on blood samples (Zhang et al., 1995; Klein et al., 1997; Fredricks and Relman, 1998). PCR-based technologies also cannot avoid sophisticated instruments such as thermal cycler and fluorescent devices, making them not suitable for point of care testing (POCT).

**Figure 1:**
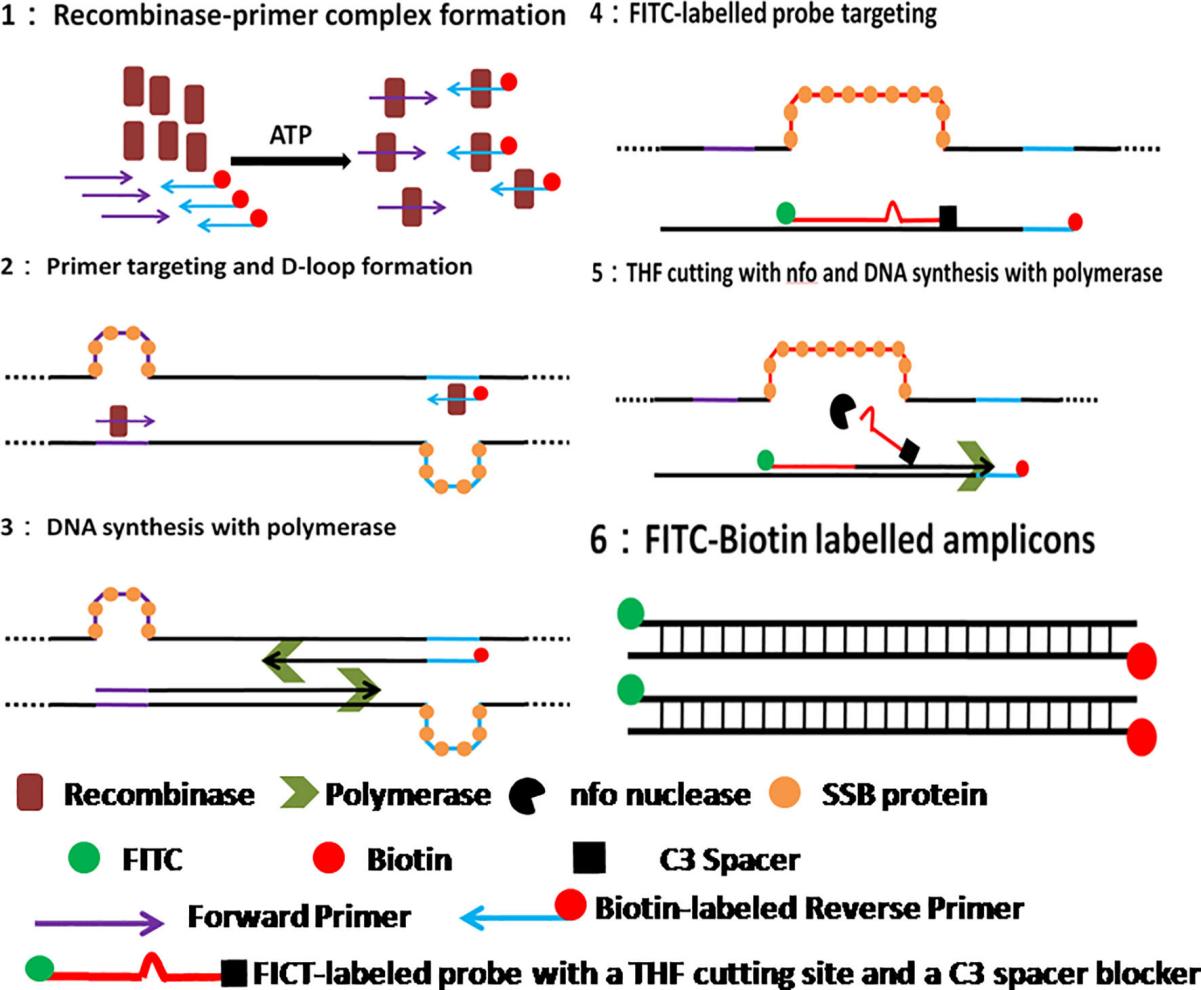
Schematic of RAA reaction. 1. Recombinase-primer complex scans the duplex DNA and locates homologous sequence; 2. D-loop structure formation, with one chain hybridizing with primer and the other chain being stabilized by SSB protein. 3. Recombinase breaks away for the next round RAA reaction, leaving the free 3’-OH of primer behind for the initiation of DNA synthesis with Polymerase; 4. RAA probe labeled with 5’-FITC and 3’-C3 spacer hybridizes target DNA and forms double stranded structure; 5. Nfo nucleases cleaves probe at the THF cutting site, leaving a free 3’-OH for DNA synthesis by Polymerase; 6. RAA amplicon formation, with a 5’-FITC group and a 3’-biotin group.

**Figure 2:**
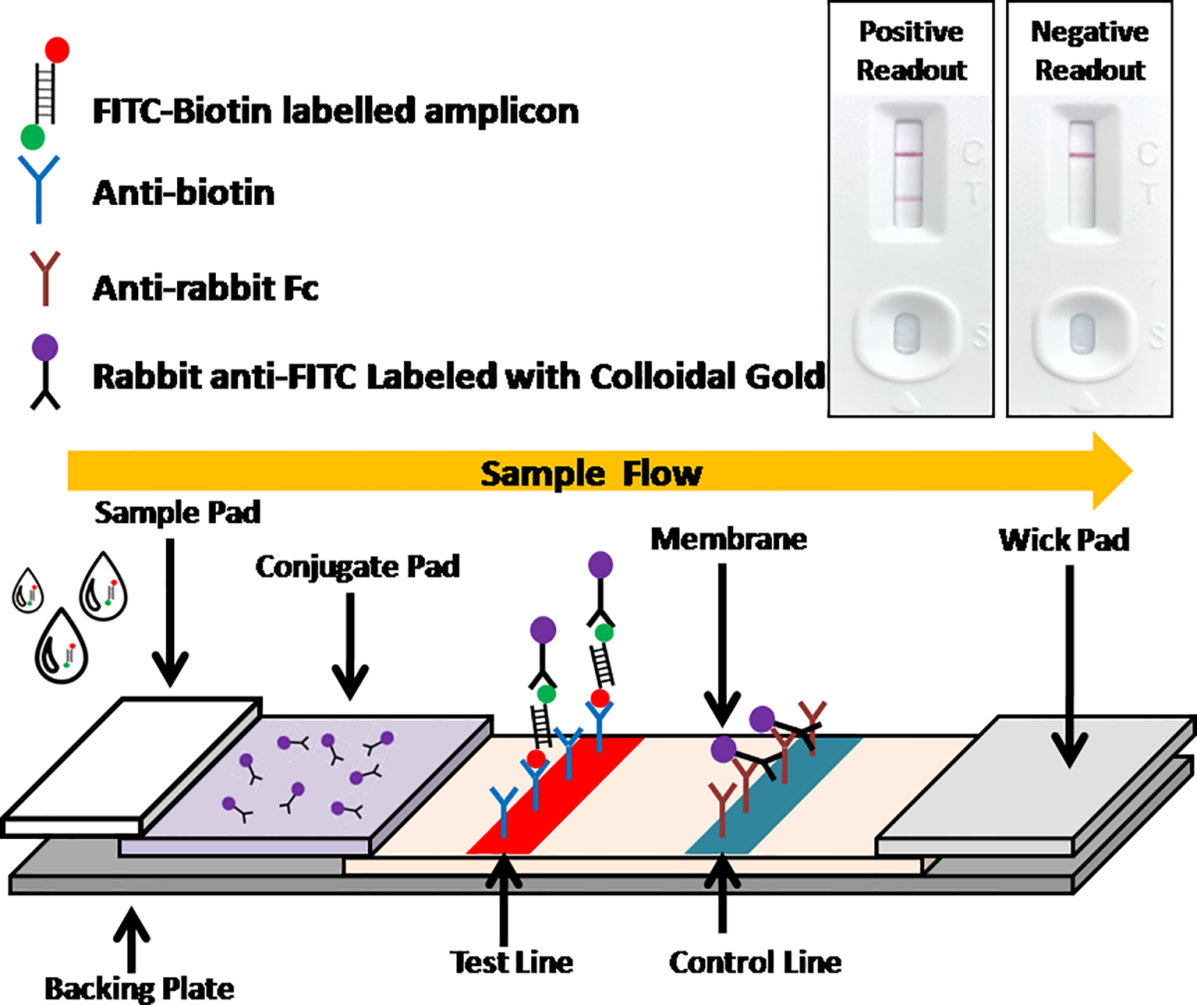
Schematic of LFA visual readout. Biotin-FITC-labeled RAA amplificons firstly conjugate with colloidal gold labeled anti-FITC antibodies. As the conjugate flowing, streptavidins can capture the conjugate at test line, while anti-mouse antibodies can capture free excess colloidal gold labeled anti-FITC antibodies at control line. Visual bands at both test line and control lines indicates a positive readout while only a single band at control line indicates a negative readout.

Recently, the isothermal amplification techniques have become an alternative way to PCR for ASFV molecular detection, including polymerase cross-linking spiral reaction (PCLSR) (Woźniakowski et al., 2017), isothermal cross-priming amplification (CPA) (Frączyk et al., 2016; Gao et al., 2018), loop-mediated isothermal amplification (LAMP) (James et al., 2010; Atuhaire et al., 2014; Wang D. et al., 2020), and recombinase based isothermal amplification assays (Wang et al., 2017; Miao et al., 2019; Fan et al., 2020; Zhai et al., 2020). With simple primer requirement and high amplification efficiency, recombinase based isothermal amplification assays, including recombinase polymerase amplification (RPA) and recombinase-aided amplification (RAA) display various advantages for POCT. Both RPA and RAA can be completed within 30 min at a constant temperature from 37 to 42°C under the action of recombinase, single-stranded DNA binding protein and polymerase (**Figure 1**). Combined with lateral flow assay (LFA) for readout (**Figure 2**) or portable fluorescent detection devices, RPA and RAA has been applied for many infectious diseases as a powerful in-field diagnostic tool for POCT (James and Macdonald, 2015; Daher et al., 2016; Moore and Jaykus, 2017; Lobato and O’Sullivan, 2018).

**Figure 3:**
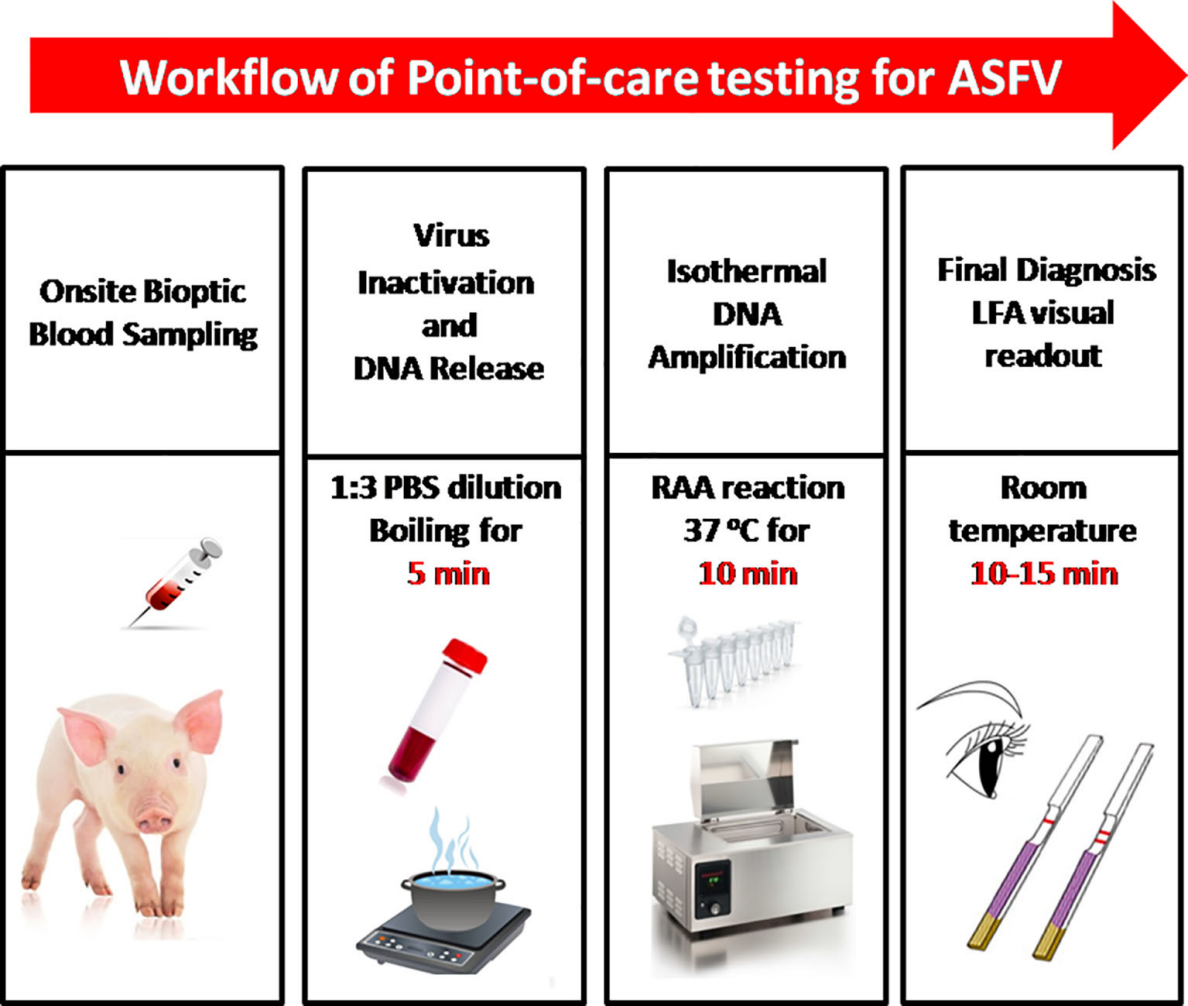
The intact point of care testing workflow for ASF from sampling to final diagnosis. Blood dilution and boiling for 5 min; RAA reaction for 10 min; LFA visual readout for 10–15 min. All work can be done within 30 min, with minimal equipment requirement.

In this study, we established a rapid RAA-LFA dignostic platform of ASFV and evaluated the sensitivity, specificity, repeatability, and stability. A simple treatment of clinical blood samples for virus inactivation and nucleic acids release was also investigated. Blood samples can be directly used for RAA-LFA after simply boiled, without the need to extract viral DNA. With minimal requirements for equipment and reagent, a complete POCT workflow for ASF from sampling to final decision within 30 min at a relative constant temperature was presented in this study (**Figure 3**).

## Materials and Methods

### Clinical Samples and Ethics Statement

A total of 37 clinical blood samples were collected from a pig farm that suffered from an ASF epidemic in Henan, China. Blood sampling was performed under licenses granted by Henan Academy of Agricultural Sciences (Approval number SYXK 2014-0007) with the animal welfare guidelines of the Institutional Animal Care and Use Committee. All sample treatments were performed at the infected farm, strictly in accordance with the standard operation for ASFV by OIE.

Other viral cell cultures used in this study, including classical swine fever virus (CSFV) Shimen strain, porcine reproductive and respiratory syndrome virus (PRRSV) HN07-1 strain, porcine epidemic diarrhea virus (PEDV) Hubei2016 strain, pseudorabies virus (PRV) HeNLH/2017 strain, and porcine circovirus 2 (PCV 2) HN-LB-2016 strain, are provided by Key Laboratory of Animal Immunology, Henan Academy of Agricultural Sciences.

### Nucleic Acid

Primers, probe, and a standard plasmid pUC57-p72 that contains ASFV Pig/HLJ/2018 strain p72-encoding gene *B646L* (GenBank: MK333180.1) used in this study were all synthesized by Sangon Biotech (Shanghai, China).

Viral DNA from clinical blood samples were extracted with MiniBEST Viral RNA/DNA Extraction Kit Ver.5.0 (Takara, Dalian, China). Briefly, 200 μl whole blood was used for extraction procedure according to manufacturer’s instruction. The total viral genomic DNA was eluted with 50 μl DEPC water and stored at -80°C for further PCR and RAA assays in this study.

### OIE Recommended PCR

According to OIE Terrestrial Manual 2019 (chapter 3.8.1 African swine fever), the recommanded PCR was performed with some modifications. A 25 μl PCR reaction contains 2×ExTaq 12.5 μl, 10 μM forward and reverse primer 0.5 μl each (OIEPCR-F and OIEPCR-R, **Table 1**), double distilled water 9 μl and DNA template 2.5 μl. The following thermal programs were: one cycle at 95°C for 10 min; 40 cycles at 95°C for 15 s, 62°C for 30 s and 72°C for 30 s; one cycle at 72°C for 7 min. PCR results were analyzed by agarose gel electrophoresis.

**Table 1:** Primer and probe sequences used in this study.

Oligo Name	Sequence
OIEPCR-F	5’-AGTTATGGGAAACCCGACCC-3’
OIEPCR-R	5’-CCCTGAATCGGAGCATCCT-3’
RAA-1F	5’-CCCGTTACGTATCCGATCACATTACCTATT-3’
RAA-2F	5’-TCAAAGTTCTGCAGCTCTTACATACCCTTCC-3’
RAA-3F	5’-TTCTGCAGCTCTTACATACCCTTCCACTAC-3’
RAA-4F	5’-TCTTACATACCCTTCCACTACGGAGGCAAT-3’
RAA-1R	5’-GTTAATAGCAGATGCCGATACCACAAGATCAG-3’
RAA-2R	5’-CGATACCACAAGATCAGCCGTAGTGATAGA-3’
RAALFA-F	5’-CCCGTTACGTATCCGATCACATTACCTATT-3’
RAALFA-R	5’-biotin-CGATACCACAAGATCAGCCGTAGTGATAGA-3’
RAALFA-Probe	5’-FITC-CAATGCGATTAAAACCCCCGATGATCCGGGTGCGA[THF]TGATGATTACCTTTGCT-C3-3’

### Quantitative Real-Time PCR

A commercial ASFV nucleic acid fluorescence PCR detection kit (MingRiDa, Beijing, China), approved by the Ministry of Agriculture and Rural Affairs of the People’s Republic of China (010688870), was used for quantitative real-time PCR (qPCR). A 25 μl qPCR reaction contains 20 μl fluorescent PCR reaction mix and 5 μl DNA template. Amplification was performed using an ABI 7500 thermocycler (Life Technologies, USA) with thermal profile as follows: one cycle at 50°C for 2 min, one cycle at 95°C for 3 min, and 40 cycles at 95°C for 10 s and 60°C for 30 s. The fluorescence signal was collected in FAM channel at the end of each cycle. It was determined positive if Ct value was less than 40 with a sigmoid-shaped amplification curve. It was determined negative if Ct value was reported as undetermined with fluorescent signal maintained at background level.

### Basic RAA Assay

RAA primers were designed in conserved regions of ASFV Pig/HLJ/2018 strain p72-encoding gene *B646L*. Four candidate forward primers (RAA-1F, RAA-2F, RAA-3F, and RAA-4F) and two candidate reverse primers (RAA-1R and RAA-2R) were screened in pairs for the best primer combination (**Table 1**). Basic RAA assay was performed at 37°C in a water bath for 30 min with basic RAA kit (Zhongce, Hangzhou, China). A 50 μl volume RAA reaction mixture included rehydration buffer (Buffer A) 41.5 μl, 20 μM forward and reverse primer 1 μl each, DNA template 4 μl and 280 mM MgOAc (Buffer B) 2.5 μl. Once RAA was completed, 50 μl DNA extraction regent (phenol:chloroform:isopropanol=25:24:1, v/v/v) was mixed with 50 μl RAA reaction and centrifuged at 10,000 rpm for 15 min. The supernatant that contained RAA amplicon was then analyzed by agarose gel electrophoresis.

### RAA Visual Readout With Lateral Flow Assay

RAA visual readout with LFA was performed with RAA nfo kit (Zhongce, Hanhzhou, China) and RAA lateral flow dipstick (Jishi, Zhengzhou, China). Primers and probe used in RAA-LFA (**Table 1**) included a common forward primer (LFA-F), a 5’-biotin-labeled reverse primer (LFA-R) and a FITC-labeled probe (LFA-probe). A 50 μl volume RAA reaction mixture included rehydration buffer (Buffer A) 40.9 μl, 20 μM forward and reverse primer 1 μl each, 10 μM probe 0.6 μl, DNA template 4 μl and 280 mM MgOAc (Buffer B) 2.5 μl. Optimal reaction temperature and time for RAA-LFA were achieved by individually performing RAA reaction at constant temperatures (room temperature, 37 or 41°C) in a water bath for 5, 10, or 15 min. After amplification, 20 μl RAA reaction was then immediately diluted with 80 μl PBST (1 x phosphate buffered saline with 0.1% Tween-20) and added to RAA lateral flow dipstick for visual readout after 10–15 min development at room temperature.

With optimal reaction condition, the sensitivity of RAA-LFA was evaluated. Decimal serial dilutions of pUC57-p72 from 10^8^ to 1 copies/μl were assayed by RAA-LFA and compared with OIE recommended PCR and commercial qPCR kit. In addition, the specificity of RAA-LFA were estimated using cell cultures of common porcine diseases, including CSFV, PRRSV, PEDV, PRV, and PCV 2.

The repeatability and stability of RAA-LFA were also evaluated. The experimental materials used for sensitivity and specificity assays were stored under the required conditions. RAA regents, primers, probe, and standard plasmid pUC57-p72 decimal serial dilutions were stored at -20°C. RAA lateral flow dipsticks were stored at room temperature under drying condition. Considering the shelf-life of RAA regents was one year, the repeatability and stability assay of RAA-LFA was performed at 0, 4, 8, 12 months after sensitivity assay, in testing 10^5^ copies/μl (strong positive sample, +++), 10^3^ copies/μl (moderate positive sample, ++), 10 copies/μl (weak positive sample, +) of standard plasmid pUC57-p72 and double distilled water (negative sample, -). All tests at each time point were repeated three times and the relative optical density (ROD) values of lateral flow dipstick’s test lines were recorded by TSR3000 membrane strip reader (BioDot manufactures, USA). Coefficient of variations (Cv) values of RAA-LFA in testing each sample were individually calculated as the ratio of standard deviation to average value among four experiments at each time point.

### Treatment of Blood Sample for Virus Inactivation and Nucleic Acid Release

To achieve both virus inactivation and nucleic acids release purposes, 2-fold serial dilutions of an ASFV positive whole blood sample were prepared with PBS and then heated in boiling water for 5 min. Optimal dilution was determined by basic RAA and compared with OIE recommended PCR. In addition, a total of 17 clinical blood samples were directly assayed with RAA-LFA after dilution and boiling, and compared with results tested with extracted viral DNAs by RAA-LFA.

## Results

### Optimization of RAA-LFA

**Figure 4:**
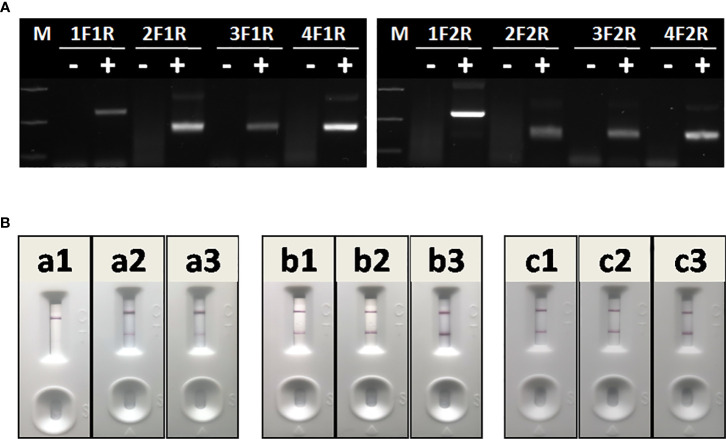
Optimization of RAA-LFA. **(A)** Optimization of primer combination. 1F1R: the combination of RAA-1F and RAA-1R; 2F1R: the combination of RAA-2F and RAA-1R. 3F1R: the combination of RAA-3F and RAA-1R; 4F1R: the combination of RAA-4F and RAA-1R. 1F2R: the combination of RAA-1F and RAA-2R; 2F2R: the combination of RAA-2F and RAA-2R. 3F1R: the combination of RAA-3F and RAA-2R; 4F2R: the combination of RAA-4F and RAA-2R. **(B)** Optimization of amplification temperature and time. a1, a2, a3: RAA was performed at room temperature for 5, 10, 15 min. b1, b2, b3: RAA was performed at 37°C for 5, 10, 15 min. c1, c2, c3: RAA was performed at 42°C for 5, 10, 15 min.

The optimal primer combination was determined by agarose gel electrophoresis of basic RAA. As shown in **Figure 4A**, among 8 primer combinations, the best RAA performance was achieved with RAA-1F and RAA-2R. Thus, these two primers were chosen as forward and reverse primers for RAA in this study.

To achieve best RAA-LFA performance, RAA reaction temperatures and times were also investigated. Three groups of RAA reactions in testing 10^5^ copies/μl standard plasmid pUC57-p72 were individually incubated at either room temperature, 37°C or 42°C for 5, 10, and 15 min. RAA performances were then analyzed with lateral flow dipstick. As shown in **Figure 4B**, RAA was not efficient at room temperature, resulting in weak positive LFA visual readout. In contrast, RAA showed good performances at both 37 and 42°C with an incubating time of 10 or 15 min. Thus, the RAA-LFA used in this study was optimized as performing RAA reaction at 37°C for 10 min, followed by LFA readout at room temperature for 10–15 min.

### Sensitivity and Specificity of RAA-LFA

**Figure 5:**
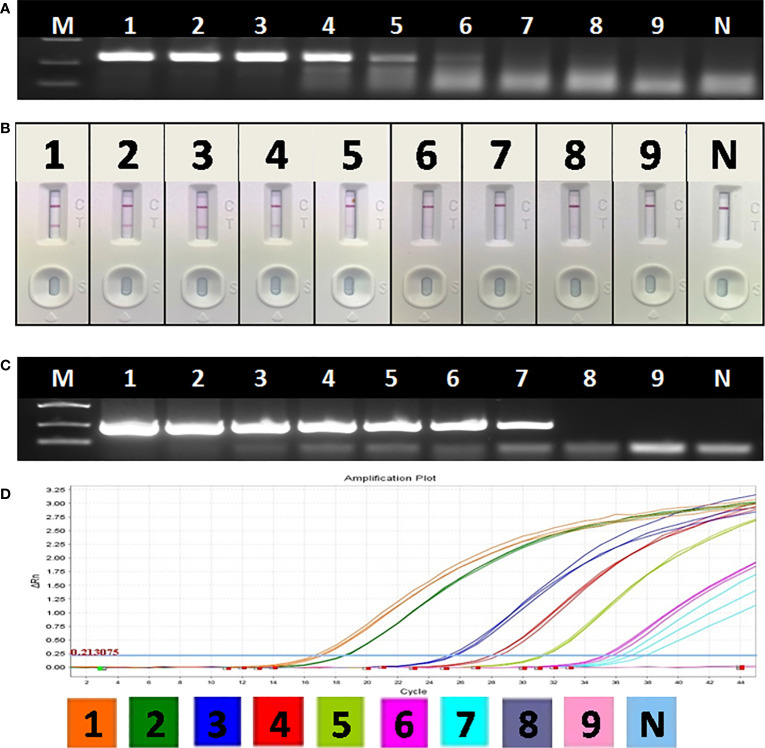
Sensitivity of OIE-recommended PCR, basic RAA and RAA-LFA for plasmid pUC57-p72. **(A)** Agarose gel electrophoresis of basic RAA. M: DL2000 DNA marker; 1–9: Decimal dilutions of plasmid pUC57-p72 from 108–100 copies/μL. N, negative control with double distilled water. **(B)** RAA-LFA visual readout. 1–9: Decimal dilutions of plasmid pUC57-p72 from 108–100 copies/μL. N, negative control with double distilled water. **(C)** Agarose gel electrophoresis of OIE-recommended PCR. M: DL2000 DNA marker; 1–9: Decimal dilutions of plasmid pUC57-p72 from 108–100 copies/μL. N, negative control with double distilled water. **(D)** qPCR amplification plot. 1–9: Decimal dilutions of plasmid pUC57-p72 from 108–100 copies/μL. N, negative control with double distilled water.

Though the detection limit of basic RAA alone was 10^3^ copies/μl (**Figure 5A**), the detection limit of RAA-LFA reached 10 copies/μl (**Figure 5B**), which is 10 fold more sensitive than both OIE recommended PCR (10^2^ copies/μl) (**Figure 5C**) and qPCR with commercial kit (10^2^ copies/μl) (**Figure 5D**).

**Figure 6:**
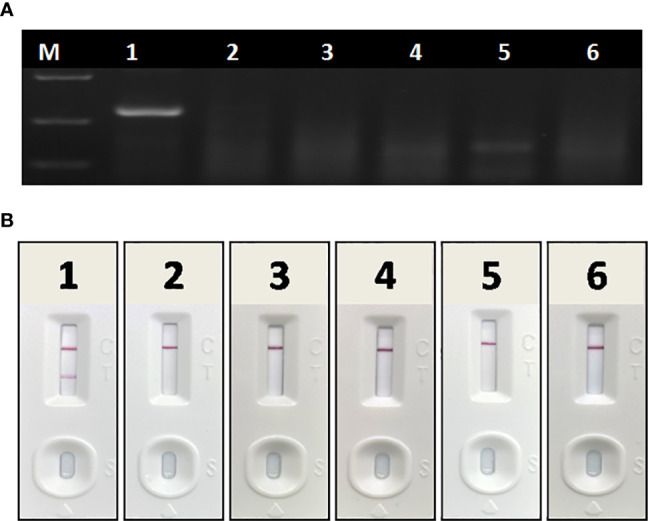
Specificity of basic RAA and RAA-LFA for common porcine diseases. **(A)** Agarose gel electrophoresis of basic RAA. M: DL 2000 DNA marker; 1: ASFV; 2: CSFV; 3: PRRSV; 4: PRV; 5: PCV 2; 6: PEDV. **(B)** RAA-LFA visual readout. 1: ASFV; 2: CSFV; 3: PRRSV; 4: PRV; 5: PCV 2; 6: PEDV.

In specificity tests, no positive readout was observed for both basic RAA and RAA-LFA in testing CSFV, PRRSV, PEDV, PRV, and PCV 2 (**Figure 6**), showing good specificity of RAA-LFA established in this study.

### Repeatability and Stability of RAA-LFA

**Figure 7:**
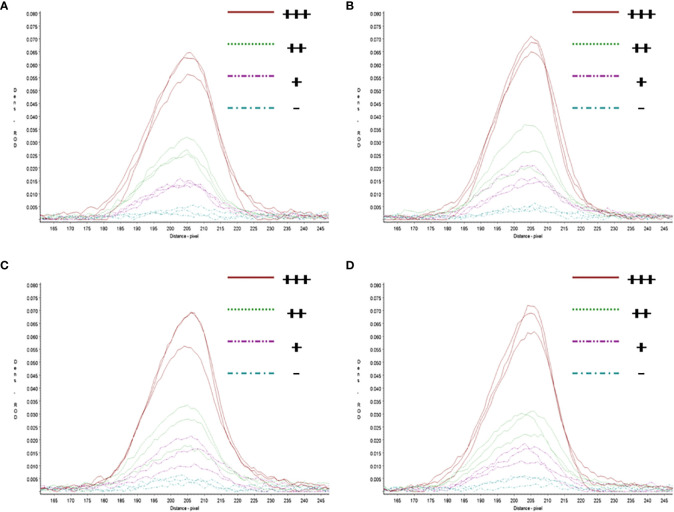
Repeatability and stability of RAA-LFA. **(A)** Screening of lateral flow dipstick’s test lines at 0 month after sensitivity and specificity assays with TSR3000 membrane strip reader. +++, strong positive sample, 10^5^ copies/μl; ++, moderate positive sample 10^3^ copies/μl; +, weak positive sample 10 copies/μl; -, negative sample, double distilled water. **(B)** Screening of lateral flow dipstick’s test lines at 4 months after sensitivity and specificity assays with TSR3000 membrane strip reader. +++, strong positive sample, 10^5^ copies/μl; ++, moderate positive sample 10^3^ copies/μl; +, weak positive sample 10 copies/μl; -, negative sample, double distilled water. **(C)** Screening of lateral flow dipstick’s test lines at 8 months after sensitivity and specificity assays with TSR3000 membrane strip reader. +++, strong positive sample, 10^5^ copies/μl; ++, moderate positive sample 10^3^ copies/μl; +, weak positive sample 10 copies/μl; -, negative sample, double distilled water. **(D)** Screening of lateral flow dipstick’s test lines at 12 months after sensitivity and specificity assays with TSR3000 membrane strip reader. +++, strong positive sample, 10^5^ copies/μl; ++, moderate positive sample 10^3^ copies/μl; +, weak positive sample 10 copies/μL; -, negative sample, double distilled water.

In repeatability and stability assay, the intensity of three repeated lateral flow dipstick’s test lines in testing strong positive sample, moderate positive sample, weak positive sample, and negative sample at 0, 4, 8, 12 months were analyzed (**Figure 7**). As shown in **Table 2**, except for the Cv value in testing marginal weak positive sample (10.45%), the Cv values of four individual experiments in testing strong positive sample (2.73%), moderate positive sample (4.01%), and negative sample (8.76%) were all less than 10%, indicating good repeatability and stability of RAA-LFA established in this study within one year.

**Table 2:** Readability and stability assay of RAA-LFA.

Time point (Month) Sample	0	4	8	12	Cv Value
	Average ROD of three repeat tests _± SD_				
10^5^ copies/μl, +++	106.2016 _± 6.9602_	106.8246 _± 3.0436_	112.7224 _± 9.8524_	109.4778 _± 9.4636_	2.73%
10^3^ copies/μl, ++	46.4175 _± 4.3735_	49.12173 _± 10.1194_	48.98073 _± 12.0557_	51.2038 _± 8.6220_	4.0%
10 copies/μl, +	25.90397 _± 2.3371_	31.94877 _± 5.7289_	30.20243 _± 8.5297_	26.21923 _± 1.7925_	10.45%
double distilled water, -	7.657333 _± 1.9936_	9.1572 _± 1.2772_	8.5984 _± 3.0125_	9.357833 _± 3.6918_	8.76%

### Evaluation of RAA-LFA in Detecting Extracted DNA From Blood Samples

**Figure 8:**
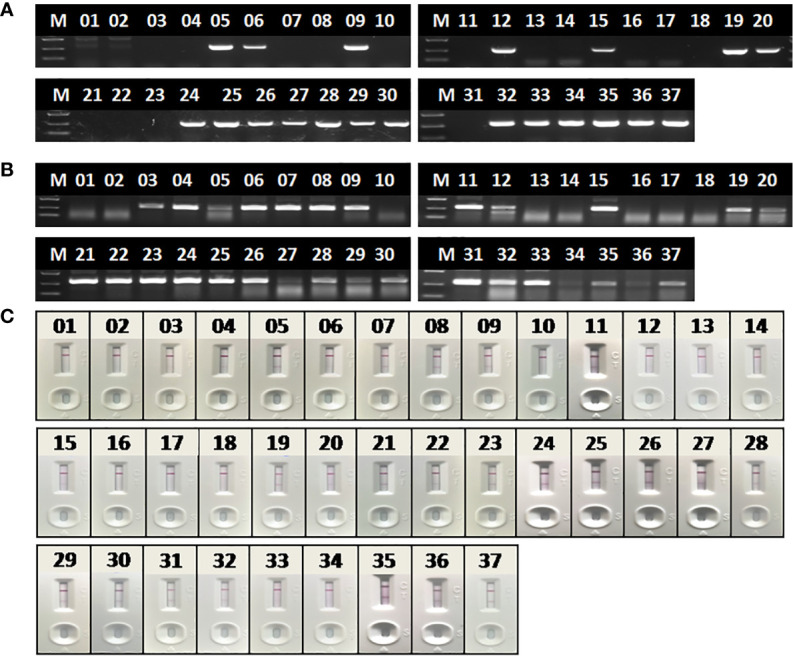
Evaluation of OIE-recommended, basic RAA and RAA-LFA in testing extracted DNA from blood samples. **(A)** Agarose gel electrophoresis of OIE-recommended PCR. M, DL2000 DNA marker; 1–37, blood sample number. **(B)** Agarose gel electrophoresis of basic RAA. M, DL2000 DNA marker; 1–37, blood sample number. **(C)** RAA-LFA visual readout. 1–37, blood sample number.

To evaluate the performance of RAA-LFA on detection of clinical samples, nucleic acids extracted from 37 blood samples were firstly tested by basic RAA, RAA-LFA and OIE recommended PCR in parallel. Initial results showed that 20 samples were tested positive and 8 samples were tested negative by all three methods. However, 9 samples were tested positive by both basic RAA and RAA-LFA (**Figures 8B, C**), but tested negative by OIE recommended PCR (**Figure 8A**).

**Figure 9:**
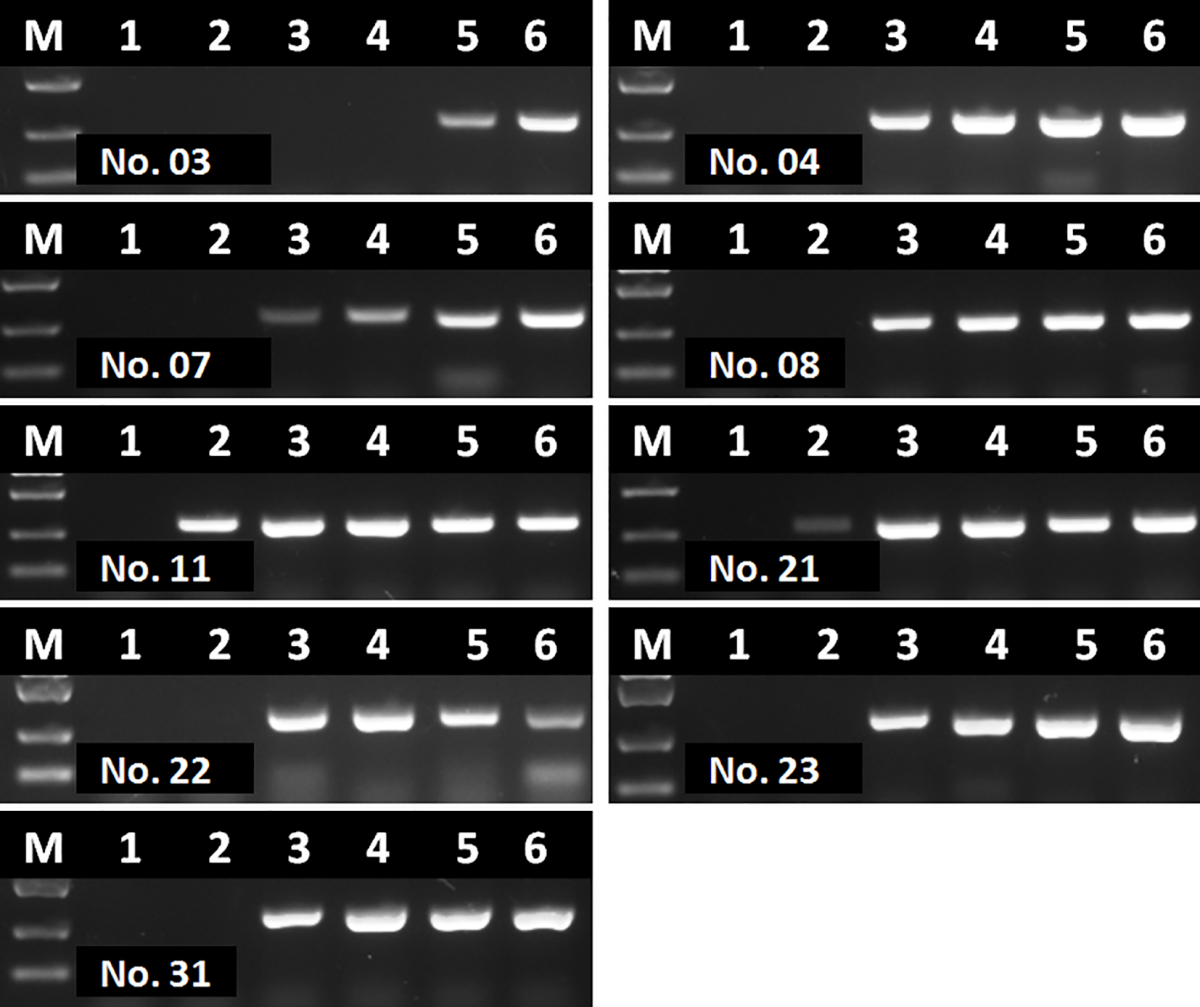
Agarose gel electrophoresis of basic RAA in testing dilutions of blood DNA with poor quality. M, DL2000 DNA maker; 1, initial DNA preparation with no dilution; 2–6, DNA dilutions with double distilled water in a ratio of 1:10, 1:20, 1:40, 1:80, and 1:160. The number of blood sample are indicated in gel electrophoresis picture, which are consistent with **Figure 8**.

Interestingly, even though extracted nucleic acids from blood samples were purified with commercial kits, some eluted DNA samples might still contain soluble colored contaminants, probably due to inappropriate blood sampling procedure. In this study, all the 9 DNA samples tested with contrary results by RAA and PCR were such cases. To verify whether these 9 DNA samples were tested false positive by RAA, or tested false negative by PCR, a serial dilutions (1/10, 1/20, 1/40, 1/80 and 1/160) of these DNA samples were prepared and tested by PCR again. Results showed that all these 9 samples were tested positive by PCR with various degrees of dilution (**Figure 9**), probably due to a reduced inhibitory effect with decreased concentration of blood contaminants. This indicated that the blood soluble colored contaminants strongly inhibited PCR but show little effect on RAA.

To verify the accuracy of RAA-LFA in testing clinical samples, a commercial ASFV qPCR kit was also used and compared with RAA-LFA and OIE recommended PCR results (**Table 3**). Taken together, RAA-LFA showed 100% positive coincident rate and 100% negative coincident rate with both OIE recommended PCR and commercial qPCR (**Table 4**).

**Table 3:** qPCR results in testing DNA samples extracted from clinical blood.

Sample No.	Ct value _± SD_	Sample No.		Ct value _± SD_	Sample No.	Ct value _± SD_
**1**	Undetermined	**14**	Undetermined		**27**	28.84 _± 0.22_
**2**	Undetermined	**15**	20.14 _± 0.18_		**28**	21.41 _± 0.056_
**3**	20.15 _± 0.02_	**16**	Undetermined		**28**	28.03 _± 0.15_
**4**	23.92 _± 0.33_	**17**	Undetermined		**30**	24.14 _± 0.26_
**5**	23.90 _± 0.58_	**18**	Undetermined		**31**	19.07 _± 0.02_
**6**	21.56 _± 0.14_	**19**	21.47 _± 0.90_		**32**	25.23 _± 0.06_
**7**	20.43 _± 0.07_	**20**	28.47 _± 0.26_		**33**	20.11 _± 0.15_
**8**	18.99 _± 0.07_	**21**	18.49 _± 0.01_		**34**	21.42 _± 0.10_
**9**	25.02 _± 0.03_	**22**	24.65 _± 0.01_		**35**	18.96 _± 0.05_
**10**	Undetermined	**23**	19.63 _± 0.24_		**36**	20.07 _± 0.34_
**11**	19.65 _± 0.11_	**24**	22.78 _± 0.02_		**37**	19.46 _± 0.10_
**12**	24.46 _± 0.21_	**25**	21.59 _± 0.13_			
**13**	Undetermined	**26**	27.10 _± 0.20_			

**Table 4:** Comparison of RAA-LFA with OIE recommended PCR and Commercial ASFV qPCR kit.

RAA-LFA	OIE recommeded PCR		Commercial ASFV qPCR kit	
+	−	+	−
**+**	29	0	29	0
**−**	0	8	0	8

### Blood Significantly Inhibits PCR, but Slightly Inhibits RAA

**Figure 10:**
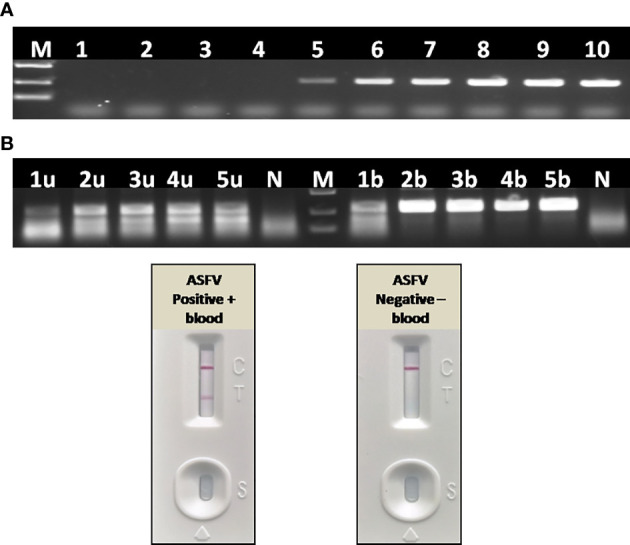
Effect of blood on the performance of PCR and RAA. **(A)** Agarose gel electrophoresis of PCR on blood samples. M, DL2000 DNA marker; 1–10, ASFV positive blood dilutions with double distilled water in a ratio of 1:2, 1:4, 1:8, 1:16, 1:32, 1:64, 1:128, 1:256, 1:512, and 1:1,024. **(B)** Agarose gel electrophoresis of RAA on blood samples. M, DL2000 DNA marker; N, negative control with double distilled water; 1u–5u, ASFV positive blood dilutions with double distilled water in a ratio of 1:2, 1:4, 1:8, 1:16, 1:32, without boiling and directly used for RAA; 1b–5b, ASFV positive blood dilutions with double distilled water in a ratio of 1:2, 1:4, 1:8, 1:16, 1:32, boiling for 5 min and used for RAA. **(C)** LFA-RAA for a positive blood samples and a negative one. Blood samples were diluted with PBS in a ratio of 1:3 and boiled for 5 min, follow by RAA reaction. Correct LFA readout indicate blood treatment used in this study was applicable for RAA isothermal amplification and LFA visual readout.

To further confirm the inhibitory effect of blood on PCR, as well as to establish a simple and rapid blood sample treatment procedure for RAA, a positive blood sample was diluted with PBS in a two-fold ratio from 1:2 to 1:16. For virus inactivation and nucleic acids release, the effect of boiling on RAA performance was also studied. Agarose gel electrophoresis showed that blood had to be diluted to at least 32–64-fold to remove the inhibitory effect on PCR (**Figure 10A**), while a 2–4-fold dilution of blood can be directly used for RAA (**Figure 10B**). In addition, the RAA performance will be further improved if blood sample was boiled for 5 min with proper dilution (**Figure 10B**), probably because that heating facilitates viral DNA release from virus particles.

### Direct Detection of ASFV From Blood Samples With RAA-LFA

Without nucleic acids extraction and purification, a positive whole blood sample and a negative one were directly assayed with RAA-LFA after being diluted with PBS in a ratio of 1:3 and boiled for 5 min. Results showed that blood treated with PBS dilution and boiling was perfectly applicable for RAA isothermal amplification and LFA visual readout (**Figure 10C**). The whole procedure can be completed within 30 min, with minimal requirements for complicated regents and expensive instruments.

**Figure 11:**
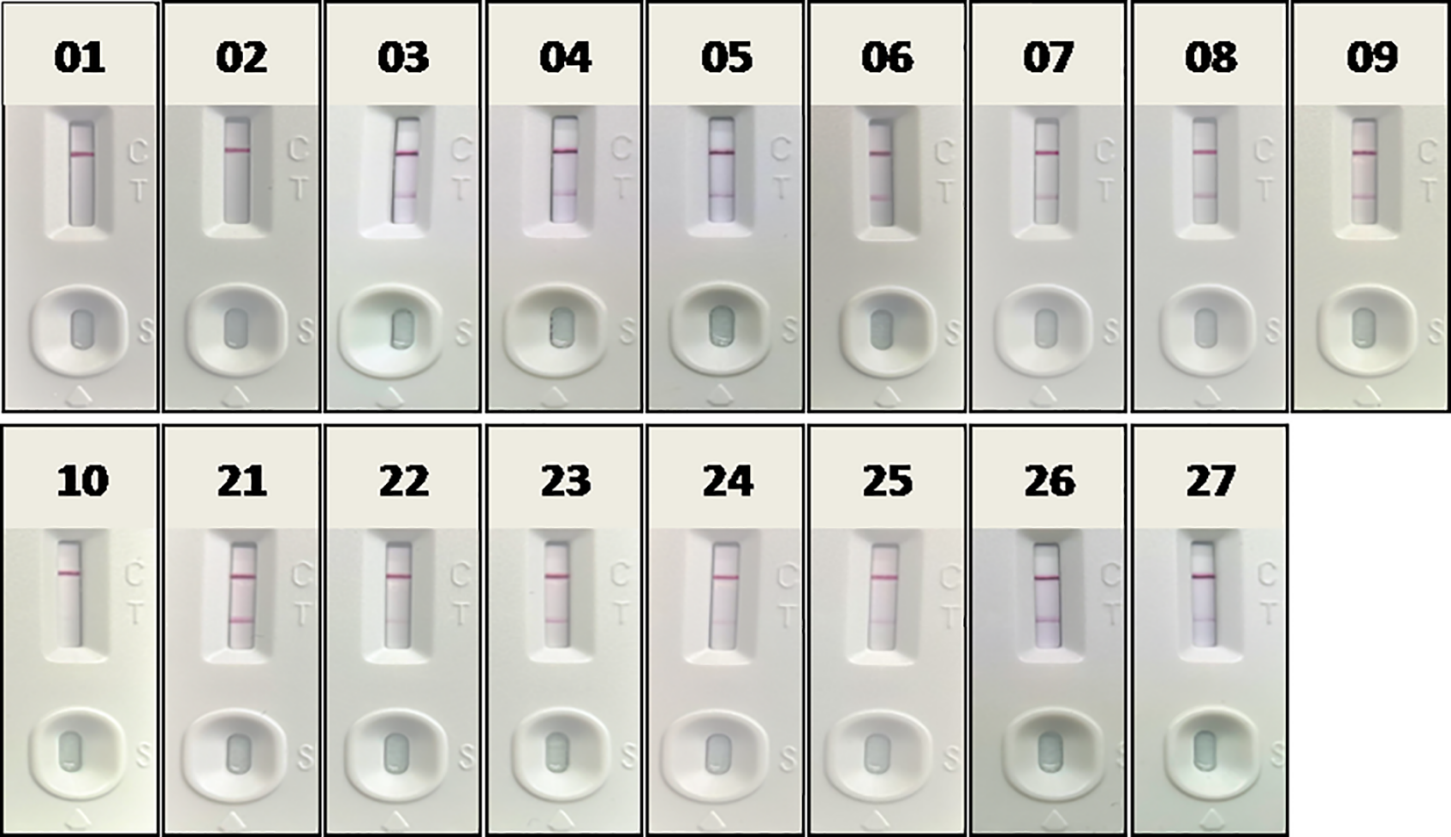
Evaluation RAA-LFA readout for detect testing blood samples without DNA extraction. The number of blood sample are indicated in gel electrophoresis picture, which are consistent with **Figures 8** and **9**.

Fourteen positive whole blood samples and three negative ones, which had been confirmed in section 3.2 by RAA-LFA, OIE recommended PCR and qPCR, were also direct tested by RAA-LFA after being 1:3 diluted with PBS and boiled for 5 min without viral DNA extraction. Results showed both 100% positive and negative coincident rates with former results (**Figure 11**).

## Discussion

In this study, we established the RAA-LFA for ASFV with high sensitivity and good specificity. The detection limit in testing synthesized plasmids reached 10 copies/μl, which is 10-fold more sensitive than OIE recommended PCR and commercial qPCR tested in parallel. In addition, no positive band was observed in testing CSFV, PCV 2, PRRSV, PEDV, and PRV by RAA-LFA. The RAA reaction can be completed at constant 37°C just in a water bath in for 10 min, followed by LFA visual readout at room temperature for 10–15 min, avoiding expensive thermal cycling and fluorescent instruments. Even though an initial evaluation of RAA-LFA and PCR on extracted DNAs from clinical blood samples showed unexpected coincident rate, we confirmed it was due to the false negative results given by PCR with some low-quality DNA preparation cases. A later PCR with serial dilutions of these low-quality DNAs preparation showed all of them were ASFV positive. Many studies have confirmed the presence of PCR-inhibitory substances in blood, such as heme, leukocyte DNA and immunoglobulin G. Single-stranded DNA binding protein gp32, one of the key enzymes involved in RAA reaction, has been shown the ability to reduce the inhibitory effects of hemoglobin and lactoferrin on polymerase activity (Akane et al., 1994; Al-Soud and Rådström, 2001). This may be a potential reason for different performances of RAA and PCR in detecting blood samples.

In combination with either portable devices for fluorescence detection or with lateral flow assay for visual readout, there has been studies using recombinase based isothermal amplification assays for POCT of ASF diagnosis (Miao et al., 2019; Zhai et al., 2020). However, all published RAA/RPA protocols to date start with the extraction of viral genomic DNA with commercial nucleic acids extraction kit. This is a considerable cost in sample preparation and also needs inconvenient and expensive instruments such as centrifuge and automatic DNA extraction machine. Considering genomic DNA of ASFV is detectable in the quite early infection stage and very stable in blood of infected animals, it is significant and possible to develop a blood sample treatment procedure compatible with RAA-LFA for POCT of ASF diagnosis, avoiding the extraction of viral DNA. In this study, we verified some components in blood samples greatly inhibited PCR performance, but has little effect on RAA. Inhibitory effect can be eliminated when blood was diluted at least 32–64-fold for directly PCR, while only a 2–4-fold dilution of blood was suitable for directly RAA, indicating RAA is a better choice that PCR when blood is used as detecting sample.

In addition, boiling was not only a reliable strategy for virus inactivation, but was also essential for direct RAA with blood. We found an improved performance when diluted blood samples was boiled, probably due to a better viral genomic DNA release from virus particles. With PBS dilution and boiling for 5 min, blood can be directly used for RAA-LFA, with 100% coincident rate with results given by RAA-LFA with extracted DNA.

## Conclusion

Taken together, we established a sensitive, specific, and rapid POCT protocol of ASF diagnosis, including a blood sample treatment procedure with dilution and boiling, an isothermal amplification with RAA and visual readout with LFA. Besides common advantages shared by other POCT methods, this protocol also meets the demands for on-site virus inactivation and bioptic purposes, providing a good choice for screening and surveillance of ASF in the future.

## Data Availability Statement

The original contributions presented in the study are included in the article/supplementary material. Further inquiries can be directed to the corresponding author.

## Ethics Statement

The animal study was reviewed and approved by Henan Academy of Agricultural Sciences (Approval number SYXK 2014-0007).

## Author Contributions

All authors contributed to the article and approved the submitted version. YZ, JG and GZ designed the research and analyzed the data. JG, GX, RD and GZ provided resources. YZ, QL, DL, LW and XW performed the experiments and wrote the manuscript.

## Funding

This work was supported by grants from the National Key Research and Development Program of China (2016YFD0500701), the Earmarked Fund for Modern Agro-Industry Technology Research System of China (CARS-35), the Special Fund for Henan Agriculture Research System (S2012-06-02), and the Key Scientific and Technological Research Projects of Henan Province (192102110007).

## Conflict of Interest

The authors declare that the research was conducted in the absence of any commercial or financial relationships that could be construed as a potential conflict of interest.
